# Enhanced Toughness of High-Entropy (Ti_0.2_Zr_0.2_Hf_0.2_Nb_0.2_Ta_0.2_)C Ceramics with SiC Whiskers by High-Pressure and High-Temperature Sintering

**DOI:** 10.3390/ma18071655

**Published:** 2025-04-04

**Authors:** Hao Li, Zhenxing Yang, Min Lian, Shuailing Ma, Wei Li, Xinmiao Wei, Xingbin Zhao, Yilong Pan, Yunfeng She, Lingyan Dang, Bao Yuan, Tian Cui

**Affiliations:** 1 School of Physical Science and Technology, Institute of High-Pressure Physics, Ningbo University, Ningbo 315211, China; lhao0224@163.com (H.L.); mashuailing@nbu.edu.cn (S.M.); 13404307481@163.com (X.W.); zhaoxingbin@nbu.edu.cn (X.Z.); panyilong@nbu.edu.cn (Y.P.); patience150@163.com (Y.S.); 2 College of Science, Hebei North University, Zhangjiakou 075000, China; yangzhenxing2017@163.com (Z.Y.); danglyhebeinu@163.com (L.D.); 3 Department of Mechanical and Materials Engineering, University of Alabama at Birmingham, Birmingham, AL 35294, USA; wli3@uab.edu; 4 Spallation Neutron Source Science Center, Dongguan 523803, China; yuanbao@ihep.ac.cn

**Keywords:** high pressure and high temperature, high-entropy carbide ceramics, silicon carbide whiskers, fracture toughness

## Abstract

High-entropy ceramics (HECs) have garnered considerable interest due to their exceptional mechanical properties and high-temperature stability. Nevertheless, their inherent brittleness significantly restricts industrial applications, posing a challenge to improving toughness without compromising hardness. This study investigates the role of SiC whiskers (SiCw) in simultaneously suppressing grain growth and enhancing the toughness of high-entropy (Ti_0.2_Zr_0.2_Hf_0.2_Nb_0.2_Ta_0.2_)C (HEC) composites, while maintaining high hardness during high-pressure high-temperature (HPHT) sintering. HEC-SiCw composites were fabricated via HPHT (P = 5 GPa, T = 2000 °C), with SiCw contents ranging from 0 to 40 mol%. As the SiCw content increased, the growth of HEC grains was inhibited, and the fracture toughness progressively rose to a peak value (K_IC_ = 9.4 ± 1.2 MPa·m^1/2^), representing an increase of approximately 184% compared to that of pure HEC, while Vickers hardness remained stable at 26 GPa. The enhancement in fracture toughness is attributed to the heterogeneous grain distribution and robust grain boundary strength, which facilitated a synergistic combination of transgranular and intergranular fracture mechanisms. These mechanisms induced crack deflection and whisker pull-out, effectively dissipating fracture energy and impeding crack propagation, thereby enhancing toughness. This study presents a novel approach to simultaneously refine grain size and improve toughness in HECs through HPHT processing, providing valuable insights for the development of next-generation ceramic composites.

## 1. Introduction

High-entropy transition metal carbide ceramics (HECs) have garnered significant attention due to their exceptional properties, including high hardness, low thermal conductivity, and excellent resistance to high temperatures and wear. Structurally, HECs are unique solid solutions composed of alternating two-dimensional layers of carbon atoms and metal cations, where the metal component consists of five or more elements in equiatomic proportions. The substantial lattice distortion inherent in these materials underpins their superior properties, rendering them highly promising for applications in extreme environments such as aerospace, nuclear energy, and high-speed cutting and machining [1,2,3]. However, a critical limitation of HECs is their poor toughness, which significantly reduces their service life and restricts their practical application scope. This challenge stems from the inherent trade-off between hardness and toughness, wherein materials with higher hardness typically exhibit lower toughness. The strong covalent bonding in ceramics generates significant lattice resistance, known as the Peierls–Nabarro (P–N) force [4], which impedes dislocation slip during fracture, limiting energy dissipation and resulting in brittle failure and low toughness. Consequently, enhancing the toughness of HECs while preserving their high hardness remains a challenge.

Common toughening strategies for ceramic materials include the incorporation of a second phase and grain refinement. Research indicates that high-density grain boundaries, leveraging the Hall–Petch effect, effectively pin dislocation motion, thereby enhancing hardness [5]. Simultaneously, intergranular fracture induces crack deflection, which improves toughness. Grain refinement has been shown to concurrently increase both strength and toughness. However, in conventional sintering processes, elevated temperatures accelerate atomic diffusion, leading to grain coarsening in HECs, which detrimentally impacts hardness and fracture toughness [6]. For instance, (Ti, Zr, Nb, Ta, Mo)C ceramics sintered at 2000 °C via spark plasma sintering (SPS) exhibited coarse grains (3.6 ± 1.4 μm) and a low fracture toughness (2.25 MPa·m^1/2^) [7]. Thus, reducing grain size and enhancing fracture toughness in HECs have become focal points of research. The incorporation of a second phase has been demonstrated to suppress grain growth, offering a simple and efficient approach suitable for industrial applications. However, compositing with lower-hardness materials, while improving toughness, often compromises hardness and thermal stability. Therefore, integrating high-hardness materials with a structure compatible with HECs emerges as an effective strategy.

Silicon carbide (SiC), sharing a similar crystal structure with HECs and possessing high hardness, stands out as an optimal second-phase candidate. Introducing SiC into HECs enables grain refinement while inducing lattice distortion and crack deflection, potentially achieving a balance between hardness and toughness [8]. For example, (Ti, Zr, Nb, Ta, Mo)C reinforced with 20 vol% SiC whiskers (SiCw) exhibited a refined grain size (0.42 μm) and enhanced fracture toughness (7.2 MPa·m^1/2^), surpassing the performance of HECs (grain size is 1.51 μm, fracture toughness is 3.4 MPa·m^1/2^) [9]. Nevertheless, the potential for further mechanical property enhancement diminishes as grain size increases, underscoring the need for innovative toughening approaches to meet practical application demands.

Compared to conventional sintering techniques, high-pressure high-temperature (HPHT) sintering is recognized as one of the most effective methods for fabricating materials with superior hardness, fracture toughness, and thermochemical stability [10,11,12]. By applying pressure, HPHT reduces the required sintering temperature, as pressure serves as a thermodynamic parameter that suppresses atomic diffusion and grain growth. Starting with smaller grain sizes, HPHT sintering prevents grain coarsening, facilitating the production of high-density, fine-grained HECs. For instance, B_6_O ceramics processed via hot-pressing and SPS exhibited enhanced toughness (1.3–3.1 MPa·m^1/2^) compared to those prepared by pressureless sintering [13]. Li et al. demonstrated that incorporating 1.5 wt% carbon nanotubes (CNTs) into polycrystalline diamond (PCD) via HPHT processing significantly increased fracture toughness to 9.1 MPa·m^1/2^, a 68.5% improvement over monolithic PCD [14,15]. Similarly, Wang et al. utilized one-dimensional single-walled carbon nanotube bundles (SWNTb) to fabricate B_4_C-SWNTb, achieving optimal hardness and toughness values of 30 GPa and 8.27 MPa·m^1/2^, respectively, with a toughness 3.3 times higher than that of pure B_4_C at 2 vol% SWNTb content [16]. However, to date, there are no published reports on the effects of SiCw incorporation on the sintering behavior, microstructure, and mechanical properties of HECs under HPHT conditions.

In this study, (Ti_0.2_Zr_0.2_Hf_0.2_Nb_0.2_Ta_0.2_)C-SiCw multiphase ceramics were prepared by incorporating varying SiCw contents (5, 10, 20, 30, and 40 mol%) under HPHT conditions to enhance the fracture toughness of HECs. The phase composition, microstructure, relative density, toughness, and fracture toughness of the synthesized ceramics were systematically investigated, alongside their impacts on Vickers hardness and fracture toughness. By elucidating the toughening mechanisms, this work proposes a novel approach to improving the mechanical properties of HECs, contributing to the broader application of advanced HEC-based ceramics.

## 2. Experimental

### 2.1. Materials Processing

High-entropy powders (99.95% purity, with a particle size of 0.3 μm, Jilin Changyu Advanced Materials Co., Ltd., Changchun, China) and commercially available SiC whisker powders (99% pure, with a diameter of 0.1–0.4 μm and length of approximately 18 μm, Changchun, China) were used as the initial materials in the experiment. The preparation of the precursor materials involved the utilization of mole fractions. The (Ti_0.2_Zr_0.2_Hf_0.2_Nb_0.2_Ta_0.2_)C powder was blended with SiC whiskers at various mole ratios—0%, 5%, 10%, 20%, 30%, and 40%—designated as HEC, HEC-5SiCw, HEC-10SiCw, HEC-20SiCw, HEC-30SiCw, and HEC-40SiCw, respectively. Subsequently, the mixture underwent a grinding process in an agate mortar and pestle for a duration of two hours to ensure uniform mixing. The uniformly mixed powder was then compacted into cylinders measuring 6 mm in diameter and 2.5 mm in height using a hydraulic press and assembled within a setup that included components such as graphite tubes. Following this, the high-pressure assembly was inserted into a large-volume cubic press for high-pressure high-temperature (HPHT) synthesis, supplied by Guiye Machinery Co., Ltd. (Guilin, China). The high-pressure and high-temperature sintering process is as follows: initially, the pressure within the cavity of the cubic anvil press is gradually increased to 5 GPa. While maintaining this pressure at 5 GPa, the temperature is elevated to the preset temperature (ranging from 1000 °C to 2000 °C) within a span of 30 s. Subsequently, this temperature is sustained for 15 min. After that, the temperature is decreased to room temperature within another 30-s period, and the pressure is gradually released to atmospheric pressure. Once the process curve was complete, the samples were allowed to cool naturally to room temperature, depressurized to atmospheric level, and removed from the assembly. The processed samples were then cut, ground, and polished for further analysis and to assess their properties.

### 2.2. Materials Characterization

To determine the phase composition of the synthesized HEC-SiCw monoliths, X-ray diffraction (Panalytic XRD with Mo Kα-ray diffraction, λ = 0.7093 Å, and a voltage of 60 kV, Tokyo, Japan) was employed. The microstructure of the fracture surfaces and cracks from Vickers hardness tests was examined using scanning electron microscopy (AURIGA Compact, Zeiss, Oberkochen, Germany). Energy dispersive spectroscopy (EDS) was used to quantify the elemental distribution within the HEC-SiCw phase. The grain size of the sintered material was assessed at the fracture surface through the average grain intercept method, with at least 100 HEC-SiCw grains measured using Nano Measurer software (version 1.2) to determine the average grain size. Grain size and orientation data were obtained through electron backscatter diffraction (EBSD) in conjunction with SEM (ZEISS GeminiSEM 360, Oxford C-Nano, Oxford, UK). The microhardness of the materials was evaluated using the Vickers indentation hardness tester (HMAS-D2000SMZC, Shanghai, China) with a load applied to the polished surface. The reported hardness is based on the average of 20 measurements for each sample. The Vickers hardness (Hv) was calculated using the formula Hv = 1.8544 × P/d^2^, where d is the average diagonal length of the indentation and P is the applied load (N) maintained for 10 s. The indentation fracture toughness (KIC) was determined by Vickers indentation with a 1 kgf load (≈9.8 N) and was calculated using the following formula: KIC = 0.016(E/Hv)^1/2^F/C^3/2^, where KIC is the indentation fracture toughness (MPa·m^1/2^), E is the Young’s modulus (GPa) from the nanoindentation test, Hv is the Vickers hardness (GPa), P is the applied load (N), and C is the average length of the radial cracks from the indentation center (μm) [17]. For the nanoindentation test, a load of 10 mN was applied. The test matrix was configured as a 5 × 5 array, with each indentation site separated by a distance of 50 μm. Density measurements were conducted using the Archimedes method in anhydrous ethanol, and the relative densities of the samples were calculated by dividing the measured densities by the theoretical densities derived from the rule of mixtures.

## 3. Results and Discussion

**Figure 1:**
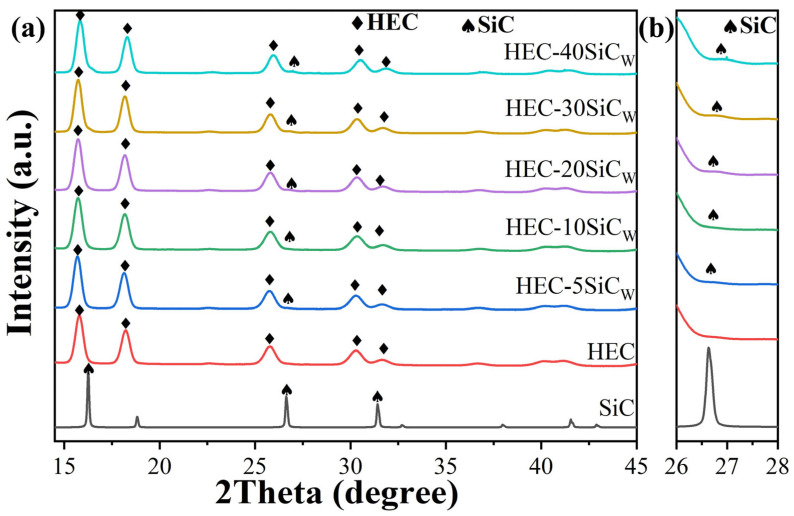
(**a**) XRD (X-ray diffraction with Mo Kα radiation λ = 0.7093 Å and voltage V = 60 kV) patterns of sintered samples with different SiCw ratios and (**b**) the corresponded enlarged XRD patterns from 2θ of 26–28°.

To explore the influence of SiCw on the mechanical properties, we conducted a series of high-pressure and high-temperature sintering experiments on HECs with varying SiCw contents. During the investigation within the temperature range of 1000–2000 °C, we determined that the performance reached its optimum at 2000 °C. Consequently, to gain a deeper understanding of the microstructure characteristics, we performed XRD tests on HECs with different SiCw contents at 2000 °C. The XRD patterns of the sintered composite samples are shown in Figure 1a. Additionally, in Figure S1, we conducted tests on the XRD patterns of HEC-1SiCW and HEC-5SiCW as a function of temperature. Notably, it is evident that the phases of the high-entropy carbides, specifically the cubic structure phase (Ti_0.2_Zr_0.2_Hf_0.2_Nb_0.2_Ta_0.2_)C, did not change after sintering. This indicates the excellent thermal stability of these high-entropy carbides under the experimental conditions. And, as the SiCw content increases, the diffraction peaks of HEC-SiCw shift toward lower angles, indicating that the incorporation of SiC results in an expansion of the HEC lattice. Furthermore, when examining the XRD patterns, it is observed that the diffraction peaks of the SiCw are extremely weak, even in the HEC-40SiCw samples. This phenomenon is likely attributed to the nanoscale size or the trace reaction products being below the detection limit. The low reference intensity ratio (RIR) value of the SiCw might also contribute, leading to a feeble signal during XRD detection. To further observe the SiCw peak, we performed magnification processing of a weak peak at 2θ = 26.6°. The peak corresponding to the (220) plane of the β-SiC phase [18] is detected in (Ti_0.2_Zr_0.2_Hf_0.2_Nb_0.2_Ta_0.2_)C-SiCw composites, as shown in Figure 1b. Figure S2 presents the X-ray diffraction (XRD) patterns of the HEC-30SiCW and HEC-40SiCW composites. Distinct diffraction peaks corresponding to silicon carbide (SiC) are prominently labeled, confirming the presence of SiC whiskers in both samples. It can be seen that the intensity of the SiCw peak gradually increases with increasing SiCw content. The XRD patterns suggest that the HPHT-sintered SiCw exhibits a textured structure, consistent with previously reported XRD results [19]. The mechanical properties of HEC-SiCw composites sintered at an initial temperature of 1000 °C are shown in Figure S3. From the results of Vickers hardness, we can observe that the performance of the material improves as the temperature rises, and it reaches its optimal state when the temperature reaches the maximum testing temperature of the machine, which is 2000 °C.

**Figure 2:**
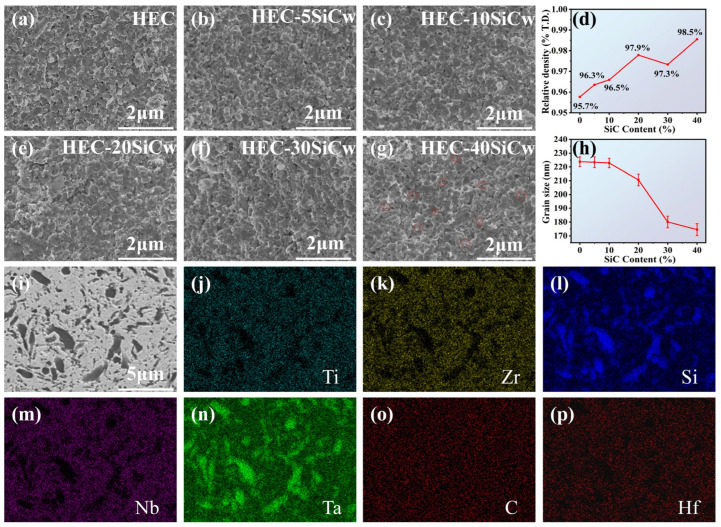
SEM images of fracture surfaces of sintered bodies and corresponding grain sizes (**a**) HEC, (**b**) HEC-5SiCw, (**c**) HEC-10SiCw, (**e**) HEC-20SiCw, (**f**) HEC-30SiCw, (**g**) HEC-40SiCw. (The SiCw is marked by a red circle in the picture). (**d**) Image of the variation law of the relative density with varying SiCw content. (**h**) Image of the pattern of variation of grain size with SiCw content. (**i**–**p**) SEM images of the polished surface of the HEC-40SiCw sample and the corresponding EDS mappings.

In order to investigate the microstructures of the composites after HPHT sintering, the microstructure of the HEC-40SiCw specimens sintered at various temperatures (1000–2000 °C) is shown in Figure S4. They are highly dense with well-bonded grains, which is in line with the trend that better performance is achieved at 2000 °C as observed in aspects such as Vickers hardness. This indicates that 2000 °C is an optimal temperature for promoting the densification and microstructure improvement of the HEC-40SiCw composites during the HPHT-sintering process. As the temperature is elevated to 1800 and 2000 °C, significant changes occur in the microstructure. Dense grains with transgranular fracture start to appear, which is in perfect agreement with the previous relative density results. This further validates that the specimens reach a high-density state at 2000 °C. Subsequently, to further explore the influence of SiCw content, specimens with different SiCw contents were sintered at this optimal temperature of 2000 °C. The cross-sectional SEM images of the HEC samples sintered with different content of SiCw at 2000 °C are shown in Figure 2a–g. It is evident that the samples exhibit high density. Figure S5 presents the results of grain size statistics, indicating that the grain size distribution of the samples ranges from 170 to 220 nm, and the grain size of these specimens is smaller than that of the pure HEC materials. Figure 2h serves as a line statistical graph based on the grain size measurements in Figure S5. In Figure 2h, as the SiCw concentration increases from 0% to 40%, the average particle size of the HEC phase decreases from 223 nm to 174 nm. These two figures clearly demonstrate how the SiCw concentration affects the grain size of the HEC phase, further elucidating the role of SiCw in shaping the microstructure of the composites.

Compared with HEC, the grain growth of the HEC phase is significantly inhibited by the addition of SiCw secondary phase particles. As shown in Figure S8, which includes (a) SEM image of the SiC whisker raw material and (b) statistical graph of the grain size, the average diameter of SiCw is 0.28 μm. The inhibition is mainly due to the “pinning” effect of SiCw on grain growth [9] and the grain suppression effect by high pressure and high temperature. The grain orientation of the specimen was investigated with EBSD in Figure S6. The specimen shows homogenous HECs grains without grain orientation. Figure 2d presents a graph of relative density values obtained for the sintered materials with varying SiCw content. The theoretical density of the high-entropy carbide phase was calculated as 9.389 g/cm^3^. The overall relative density was subsequently determined using the theoretical density of SiC (3.21 g/cm^3^). XRD analysis confirmed the absence of secondary phases, and therefore, their influence on the relative density was deemed negligible. The relative densities of the HEC, HEC-5SiCw, HEC-10SiCw, HEC-20SiCw, HEC-30SiCw, and HEC-40SiCw samples were measured to be 95.7%, 96.3%, 96.5%, 97.9%, 97.3%, and 98.5%, respectively. The addition of whiskers to ceramics is known to have positive effects on the sintering process and densification of ceramics. As the SiCw content increases, the relative density of the resulting ceramics tends to increase. The relative low hardness and fracture toughness of HECs composites is usually caused by the weak interaction between HECs and enhanced phases, which impair the strength of composites. The relative high density suggests that HECs and SiCw have a strong interaction which is difficult to dense at ambient sintering. The HPHT method gives a strong combination of two phases. In Figure 2i, the dark-colored SiC is clearly visible. This vividly demonstrates the successful synthesis of the composite material containing SiC. As shown in Figure 2j–p, the EDS images display the elemental distribution maps. Under high temperature and pressure, the homogeneous distribution of Ti, Zr, Nb, and Hf in the high-entropy carbide (HEC) phase confirms the stability of the single-phase solid-solution structure, governed by the high-entropy “cocktail effect”. EDS results reveal that in the 40SiCw composite, the atomic percentage of Si is 8.93%. This proves that SiC has indeed been incorporated into it.

**Figure 3:**
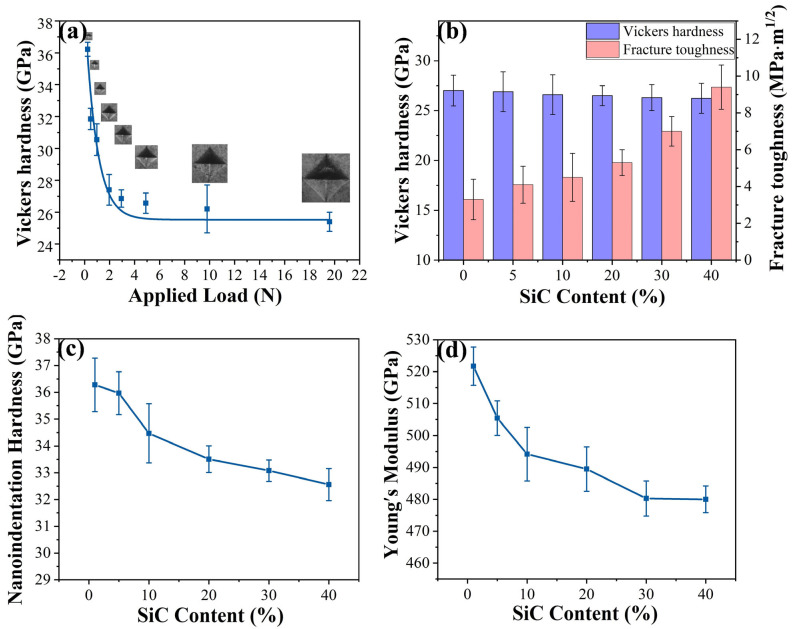
(**a**) Vickers hardness as a function of applied loads of HEC-40%SiCw. (**b**) Vickers hardness and fracture toughness with different SiCw addition ratio. (**c**) Nanoindentation hardness with different addition amounts of SiCw. (**d**) Young’s modulus as a function of the addition amount of SiCw.

**Figure 4:**
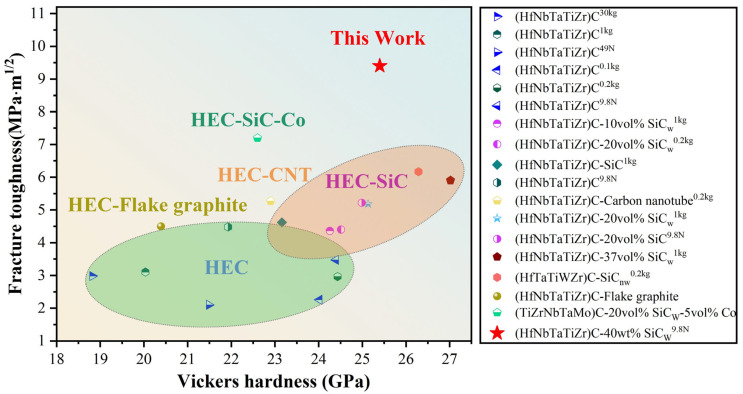
Plot of fracture toughness versus. (HfNbTaTiZr)C^30kg^ [23], (HfNbTaTiZr)C^1kg^ [24], (HfNbTaTiZr)C^49N^ [25], (HfNbTaTiZr)C^0.1kg^ [26], (HfNbTaTiZr)C^0.2kg^ [19], (HfNbTaTiZr)C^9.8N^ [27], (HfNbTaTiZr)C-10vol% SiC_w_^1kg^ [24], (HfNbTaTiZr)C-20vol% SiC_w_^0.2kg^ [22], (HfNbTaTiZr)C-SiC^1kg^ [28], (HfNbTaTiZr)C^9.8N^ [29], (HfNbTaTiZr)C-Carbon nanotube^0.2kg^ [30], (HfNbTaTiZr)C-20vol% SiC_w_^1kg^ [24], (HfNbTaTiZr)C-20vol% SiC^9.8N^ [29], (HfNbTaTiZr)C-37vol% SiC_w_^1kg^ [24], (HfTaTiWZr)C-SiC_nw_^0.2kg^ [30], (HfNbTaTiZr)C-Flake graphite [31], (TiZrNbTaMo)C-20vol% SiC_W_-5vol% Co [9].

The mechanical properties of HEC with varying SiCw content are presented in Figure 3. Figure 3a and Figure S7 shows that the Vickers hardness tends to stabilize with the applied load of 9.8 N for HEC-40SiCw specimen. Figure 3b shows the Vickers hardness of HECs with different SiCw content. The asymptotic hardness values of the HEC, HEC-5SiCw, HEC-10SiCw, HEC-20SiCw, HEC-30SiCw, and HEC-40SiCw samples were 27.0 ± 1.54 GPa, 26.9 ± 2 GPa, 26.6 ± 2.1 GPa, 26.5 ± 1 GPa, 26.3 ± 1.3 GPa, and 26.2 ± 1.5 GPa, respectively. The hardness of all samples is a little bit lower than pure HECs with the increasing content of SiCw. However, all samples exhibit similar Vickers hardness values of 26–27 GPa, due to the high hardness of SiCw, strong combination between two phases, and the fine grain size of HEC within the composites. Figure 3 shows the relationship between the SiCw ratio and (c) nanoindentation hardness and (d) Young’s modulus of samples sintered at 5 GPa and 2000 °C, where the modulus can be used to calculate toughness. Among the as-prepared samples, the HEC has the lowest fracture toughness of 3.3 ± 1.1 MPa·m^1/2^ (Figure 3b). The fracture toughness of the HEC-SiCw composites increased with the increasing SiCw content (Figure 3b). The highest fracture toughness is up to 9.4 ± 1.2 MPa·m^1/2^ for the HEC-40SiCw sample, which increases ~184% more than the unreinforced HEC. The toughness values of HEC-5SiCw, HEC-10SiCw, HEC-20SiCw, and HEC-30SiCw are 4.1 ± 1 MPa·m^1/2^, 4.5 ± 1.3 MPa·m^1/2^, 5.3 ± 0.7 MPa·m^1/2^, and 7.0 ± 0.8 MPa·m^1/2^, respectively. In general, most of the HEC composites with additives can improve the toughness about 0.73–3.8 MPa·m^1/2^ [20,21,22], including the (Ti_0.2_Zr_0.2_Hf_0.2_Nb_0.2_Ta_0.2_)C-20vol %SiCw-5vol %Co composites (Figure 4) [9]. Meanwhile, the hardness of these composites is in the range of 26–27 GPa. In addition, a handful of composites can achieve a fracture toughness of more than 4 MPa·m^1/2^ and high hardness around 24 GPa, but rarely can composites achieve high fracture toughness around 10 MPa·m^1/2^ and maintain the high hardness more than 25 GPa (Figure 4) [23,24,25,26,27,28,29,30,31]. Thus, the HEC-40SiCw sample shows almost the highest fracture toughness with high hardness more than 25 GPa. Thus, sintering by HPHT can effectively increase the toughness of HEC-SiCw composites without decreasing the hardness. Toughing the HECs with SiCw breaks the contradictory of hardness and toughness due to the simultaneously strengthening effect of SiCw.

**Figure 5:**
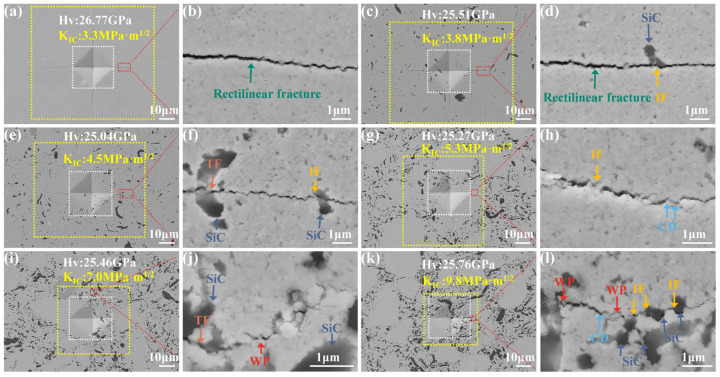
Representative SEM images of indentation morphology and surface crack propagation paths of sintered samples at 5 GPa and 2000 °C: (**a**,**b**) HEC, (**c**,**d**) HEC-5SiCw, (**e**,**f**) HEC-10SiCw, (**g**,**h**) HEC-20SiCw, (**i**,**j**) HEC-30SiCw, and (**k**,**l**) HEC-40SiCw. Arrows show the crack deflection and whisker pullout. (TF: transcrystalline fracture, WP: whisker pulling, IF: intergranular fracture, CD: crack deflection).

Vickers hardness of HEC-40%SiCw composites studied in this work as compared to related HEC materials from the literatures. HEC-CNT is the composite of carbon nanotube tougheners and high-entropy powder. HEC-SiC-Co is a (Ti, Zr, Nb, Ta, Mo)C-SiCw composite with co additive. To reveal the toughening mechanism of HEC-SiCw ceramic composites, the Vickers hardness indentation and crack extension was evaluated using the SEM images, as shown in Figure 5. Use the yellow box to select cracks in all four directions to calculate the average value of c to calculate fracture toughness. The diagonal d value of the indentation used to calculate Hv is marked by the white dashed line. There is a straight crack with relatively few cracks deflection along the cracks in HECs (Figure 5a,b). For the sintered HEC-SiCw samples, the crack deflection between the HEC matrix and SiCw can be seen from Figure 5c,d, where a transcrystalline fracture is also observed in this image. From Figure 5e–j, as the amount of SiCw increases, the cracks become progressively more tortuous. This highlights the enhanced strengthening mechanism at work. Crack deflection and intergranular fracture is particularly evident along the cracks in Figure 5k,l (highlighted by arrows). The crack deflection is especially evident at the junction between HEC and SiC grains as shown in Figure 5l. This microstructural evidence substantiates that the strategic incorporation of SiC whiskers through controlled fabrication parameters effectively induced a whisker-reinforcement mechanism, thereby contributing to enhanced mechanical strength through crack deflection and load transfer phenomena. For the HEC-SiCw ceramic composites, the toughening effect is like that of SiCw whisker-toughened ceramics, and the main toughening mechanism is crack deflection due to the hard pinning effect of the secondary SiCw [32,33,34,35,36]. With the increasing content of SiCw, more HECs and SiCw interface generate, which is beneficial for the crack deflection because it usually happens at the interface of different phases. Moreover, with the increase of its content, the main toughening mechanism is not only crack deflection, but also whisker pullout and transgranular fracture. Therefore, the main toughening mechanisms of whisker-reinforced composites are crack deflection, whisker pullout, and transgranular fracture, which become more frequent with the increase of SiCw content due to the higher density of interface between two phases.

## 4. Conclusions

In this study, HEC-SiCw composites with different SiCw contents were sintered at HPHT (P = 5 GPa, T = 2000 °C). The results demonstrated that with increasing SiCw content, the grain of the HEC is significantly inhibited due to the hard pinning effect of the secondary SiCw phase. With the increase of SiCw content from 0 to 40 mol%, the fracture toughness increased from 3.3 ± 1.1 MPa·m^1/2^ to 9.4 ± 1.2 MPa·m^1/2^, and the Vickers hardness values basically remain unchanged. Based on SEM observations, the main toughening mechanisms are found to be crack deflection and whisker pullout, which effectively consumes fracture energy and enhances toughness. This work provides a method to inhibit grain growth and enhance toughness under HPHT, which is important for the development of future robust ceramic materials.

## Data Availability

The original contributions presented in this study are included in the article/Supplementary Materials. Further inquiries can be directed to the corresponding authors.

## Supplementary Materials

The following supporting information can be downloaded at: https://www.mdpi.com/article/10.3390/ma18071655/s1, Figure S1: (a) XRD patterns obtained at HEC-1SiCw with different sintering temperatures. (b) XRD patterns obtained at HEC-5SiCw with different sintering temperatures; Figure S2: XRD patterns of HEC-30SiCw and HEC-40SiCw obtained under the conditions of 5 GPa and 2000 °C; Figure S3: (a) Vickers hardness image of HEC-1SiCw material at 1000 °C under different loads. (b) Vickers hardness images at different temperatures at 1 kg. (c) Variation of sintering density with silicon addition. (d) Variation of sintered density with sintering temperature for HEC-1SiCw; Figure S4: SEM images of fracture surfaces of sintered bodies. (HEC-40SiCw (a) 1000 °C, (b) 1200 °C, (c) 1400 °C, (d) 1600 °C, (e) 1800 °C, (f) 2000 °C); Figure S5: Grain sizes corresponding to different doping ratios of the sintered body. (a) HEC, (b) HEC-5SiCw, (c) HEC-10SiCw, (d) HEC-20SiCw, (e)HEC-30SiCw, (f) HEC-40SiCw; Figure S6: Microstructure of the sintered HEC-SiCw alloy with 40 mol% SiCw under the conditions of 5 GPa and 2000 °C: (a) SEM image, (b) phase diagram, (c) IPF; Figure S7: Under the conditions of 5 GPa and 2000 °C (a) Vickers hardness as a function of applied loads, (b) Vickers hardness of different additives at applied loads of 1 kg (10 N) and 2 kg (20 N); Figure S8: (a) SEM image of the SiC whisker raw material (b) Statistical graph of the grain size.

## References

[1] Wang Y., Csanádi T., Zhang H., Dusza J., Reece M.J., Zhang R. (2020). Enhanced Hardness in High-Entropy Carbides through Atomic Randomness. Adv. Theory Simul..

[2] Wang K., Chen L., Xu C., Zhang W., Liu Z., Wang Y., Ouyang J., Zhang X., Fu Y., Zhou Y. (2020). Microstructure and Mechanical Properties of (TiZrNbTaMo)C High-Entropy Ceramic. J. Mater. Sci. Technol..

[3] Liu P., Peng F., Liu F., Wang H., Xu C., Wang Q., Zhou X., Yin W., Yin S., Li Y. (2014). High-Pressure Preparation of Bulk Tungsten Material with near-Full Densification and High Fracture Toughness. Int. J. Refract. Met. Hard Mater..

[4] Peierls R. (1940). The size of a dislocation. Proc. Phys. Soc..

[5] An Z., Mao S., Liu Y., Yang L., Vayyala A., Wei X., Liu C., Shi C., Jin H., Liu C. (2023). Inherent and Multiple Strain Hardening Imparting Synergistic Ultrahigh Strength and Ductility in a Low Stacking Faulted Heterogeneous High-Entropy Alloy. Acta Mater..

[6] Wang X., Tao Q., Han Y., Hu Q., Cheng J., Jia H., Zhu P. (2020). TiB_2_ -Reinforced B_4_C Composites Produced by Reaction Sintering at High-Pressure and High Temperature. High Press. Res..

[7] Luo S.-C., Guo W.-M., Plucknett K., Lin H.-T. (2022). Low-Temperature Densification of High-Entropy (Ti,Zr,Nb,Ta,Mo)C—Co Composites with High Hardness and High Toughness. J. Adv. Ceram..

[8] Tu J., Wang X., Dai W., Zhang H., Liu B. (2023). Mechanical Properties and Microstructures of HPHT Sintered Polycrystalline Diamond Compacts Reinforced with SiC Whiskers. J. Mater. Res. Technol..

[9] Liu Y., Guo W.-M., Xu L., Sun S.-K., Lin H.-T. (2023). Low-Temperature Sintered (Ti, Zr, Nb, Ta, Mo)C-Based Composites Toughened with Damage-Free SiCw. J. Eur. Ceram. Soc..

[10] Wang J., Wang Z., Wang Y., Ma H., Fang S., Chen Q., Wang Y., Wang C., Chen L., Jia X. (2022). Effects of Pressure on the Phase Transformations and Mechanical Properties of 10 Mol% Mg-PSZ Sintered by HPHT. CrystEngComm.

[11] Zhang Y., Zhu Y., Li Z. (2023). Preparation and Characterization of Carbide Particle-Toughened Si–B System of High Thermostability Polycrystalline Diamond by HPHT Sintering. Materials.

[12] Laurindo Q.M.G., Rosa J.M.B., Da Silva Guimarães R., Teixeira S.R., Lima L.S., Xing Y., Filgueira M. (2024). Polycrystalline Diamond Obtained in the Diamond-Mo System with Enhanced Thermal Stability Sintered by HPHT. Int. J. Refract. Met. Hard Mater..

[13] Zhang B., Sun R., Ying P., Li P., Li Z., Gao Y., Ma M., Liu C. (2023). High-Pressure Sintering of Full-Dense B_6_O Ceramic with Excellent Hardness and Fracture Toughness. Int. J. Refract. Met. Hard Mater..

[14] Jian Q., Jiang Z., Han Y., Zhu Y., Li Z. (2020). Fabrication and Evaluation of Mechanical Properties of Polycrystalline Diamond Reinforced with Carbon-Nanotubes by HPHT Sintering. Ceram. Int..

[15] Zou Q., Wu H., Li Y., Wang X., Dai L., Luo Y. (2022). Effects of Carbon Nanotubes and Sintering Parameters on Microstructure and Properties of PCD. Diam. Relat. Mater..

[16] Wang X., Wang D., Ma S., Dong X., Rong K., You C., Wang F., Li H., Li D., Tao Q. (2023). Enhanced Toughness of Boron Carbide by Single-Wall Carbon Nanotube Bundles. Mater. Today Commun..

[17] Anstis G.R., Chantikul P., Lawn B.R., Marshall D.B. (1981). A Critical Evaluation of Indentation Techniques for Measuring Fracture Toughness: I, Direct Crack Measurements. J. Am. Ceram. Soc..

[18] Song B., Zhao B., Lu Y., Wei S., Fan B., Zhang X., Zhang R. (2018). Investigation on the Growth Mechanism of SiC Whiskers during Microwave Synthesis. Phys. Chem. Chem. Phys..

[19] Luo S.-C., Guo W.-M., Zhou Y.-Z., Plucknett K., Lin H.-T. (2021). Textured and Toughened High-Entropy (Ti_0.2_Zr_0.2_Hf_0.2_Nb_0.2_Ta_0.2_)C-SiCw Ceramics. J. Mater. Sci. Technol..

[20] Nisar A., Dolmetsch T., Paul T., Sakthivel T.S., Zhang C., Boesl B., Seal S., Agarwal A. (2022). Unveiling Enhanced Oxidation Resistance and Mechanical Integrity of Multicomponent Ultra-high Temperature Carbides. J. Am. Ceram. Soc..

[21] Zhang W., Chen L., Xu C., Lu W., Wang Y., Ouyang J., Zhou Y. (2021). Densification, Microstructure and Mechanical Properties of Multicomponent (TiZrHfNbTaMo)C Ceramic Prepared by Pressureless Sintering. J. Mater. Sci. Technol..

[22] Kombamuthu V., Ünsal H., Chlup Z., Tatarková M., Kovalčíková A., Zhukova I., Hosseini N., Hičák M., Dlouhý I., Tatarko P. (2024). Effect of SiC on Densification, Microstructure and Mechanical Properties of High Entropy Diboride (Ti_0.2_Zr_0.2_Hf_0.2_Nb_0.2_Ta_0.2_)B_2_. J. Eur. Ceram. Soc..

[23] Liu J., Xiong J., Guo Z., Zhou H., Yang T., Yang L., Zhao W. (2020). Preparation of High-Entropy (Zr_0.25_Hf_0.25_Ta_0.25_Ti_0.25_)C-Ni-Co Composite by Spark Plasma Sintering. Metall. Mater. Trans. A.

[24] Wang Z., Li Z.-T., Zhao S.-J., Wu Z.-G. (2021). High-Entropy Carbide Ceramics: A Perspective Review. Tungsten.

[25] Wei X.-F., Liu J.-X., Bao W., Qin Y., Li F., Liang Y., Xu F., Zhang G.-J. (2021). High-Entropy Carbide Ceramics with Refined Microstructure and Enhanced Thermal Conductivity by the Addition of Graphite. J. Eur. Ceram. Soc..

[26] Sun K., Yang Z., Mu R., Niu S., Wang Y., Wang D. (2021). Densification and Joining of a (HfTaZrNbTi)C High-Entropy Ceramic by Hot Pressing. J. Eur. Ceram. Soc..

[27] Feng L., Chen W., Fahrenholtz W.G., Hilmas G.E. (2021). Strength of Single-phase High-entropy Carbide Ceramics up to 2300 °C. J. Am. Ceram. Soc..

[28] Lin G., Liu J., Qin Y., Zhang G. (2022). Low-temperature Reactive Sintering of Carbon Vacant High-entropy Carbide Ceramics with In-situ Formed Silicon Carbide. J. Am. Ceram. Soc..

[29] Lu K., Liu J.-X., Wei X.-F., Bao W., Wu Y., Li F., Xu F., Zhang G.-J. (2020). Microstructures and Mechanical Properties of High-Entropy (Ti_0.2_Zr_0.2_Hf_0.2_Nb_0.2_Ta_0.2_)C Ceramics with the Addition of SiC Secondary Phase. J. Eur. Ceram. Soc..

[30] Sun J., Zhao J., Chen Y., Wang L., Yun X., Huang Z. (2022). Toughening in Low-Dimensional Nanomaterials High-Entropy Ceramic Nanocomposite. Compos. Part B Eng..

[31] Cao Z., Sun J., Meng L., Zhang K., Zhao J., Huang Z., Yun X. (2023). Progress in Densification and Toughening of High Entropy Carbide Ceramics. J. Mater. Sci. Technol..

[32] Zhao X., Shao G., Feng L., Wang H., Fan B., Lu H., Xu H., Chen D., Zhang R. (2017). ZrB_2_ -SiC_w_ Ceramic Composites Synthesized by in Situ Reaction and Spark Plasma Sintering. Int. J. Appl. Ceram. Technol..

[33] Yan T., Luo M., Chen J., Zhu H., Chai J., Niu L., Chen B., Zhu Y., Shen T. (2024). Microstructure and Mechanical Properties of High Entropy (MoTaTiVW)C_5_ Ceramics Toughened with Silicon Carbide Whisker. Ceram. Int..

[34] Rumiantseva Y.Y., Bushlya V.N., Turkevich V.Z. (2019). The Influence of SiC and Al_2_O_3_ Whiskers on the Properties of Whisker-Reinforced cBN-Based Composites. J. Superhard Mater..

[35] Zhu T., Guo W., Zhang J., Sang S., Li Y., Xie Z., Liang X., Wang H., Han Y. (2023). Synergistic Toughening Effect of SiC Whiskers and Particles in ZrO_2_–Al_2_O_3_–SiC Ceramics. Ceram. Int..

[36] Li Z., Guo R., Li L., Zheng R., Ma C. (2023). Microstructure and Fracture Toughness of SiAlCN Ceramics Toughened by SiCw or GNPs. Ceram. Int..

