# Supplementary material for: Enhanced Toughness of High-Entropy (Ti0.2Zr0.2Hf0.2Nb0.2Ta0.2)C Ceramics with SiC Whiskers by High-Pressure and High-Temperature Sintering
Source: Materials (Basel). 2025 Apr 4;18(7):1655. doi: 10.3390/ma18071655 (PMC11990707; doi:10.3390/ma18071655)
Supplement: Supplementary file 1 [file materials-18-01655-s001.zip › materials-3512593-supplementary.pdf]

Supplementary Material:

Enhanced Toughness of High-Entropy ($Ti_{0.2}Zr_{0.2}Hf_{0.2}Nb_{0.2}Ta_{0.2}$)C Ceramics with SiC Whiskers by High-Pressure and High-Temperature Sintering

Hao Li [1], Zhenxing Yang [2], Min Lian [1,*], Shuailing Ma [1], Wei Li [3], Xinmiao Wei [1], Xingbin Zhao [1], Yilong Pan [1], Yunfeng She [1], Lingyan Dang [2], Bao Yuan [4] and Tian Cui [1,*]

[1] School of Physical Science and Technology, Institute of High-Pressure Physics, Ningbo University, Ningbo 315211, China; lhao0224@163.com (H.L.); mashuailing@nbu.edu.cn (S.M.); 13404307481@163.com (X.W.); zhaoxingbin@nbu.edu.cn (X.Z.); panyilong@nbu.edu.cn (Y.P.); patience150@163.com (Y.S.)

[2] College of Science, Hebei North University, Zhangjiakou 075000, China; yangzhenxing2017@163.com (Z.Y.); danglyhebeinu@163.com (L.D.)

[3] Department of Mechanical and Materials Engineering, University of Alabama at Birmingham, Birmingham, AL 35294, USA; wli3@uab.edu

[4] Spallation Neutron Source Science Center, Dongguan 523803, China; yuanbao@ihep.ac.cn

* Correspondence: lianmin@nbu.edu.cn (M.L.); cuitian@nbu.edu.cn (T.C.)

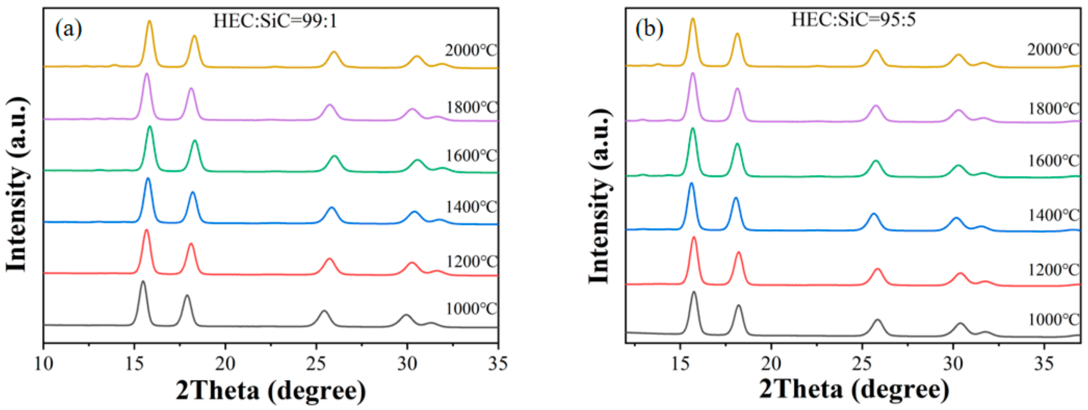

Figure S1. (a) XRD patterns obtained at HEC-1SiCw with different sintering temperatures. (b) XRD patterns obtained at HEC-5SiCw with different sintering temperatures.

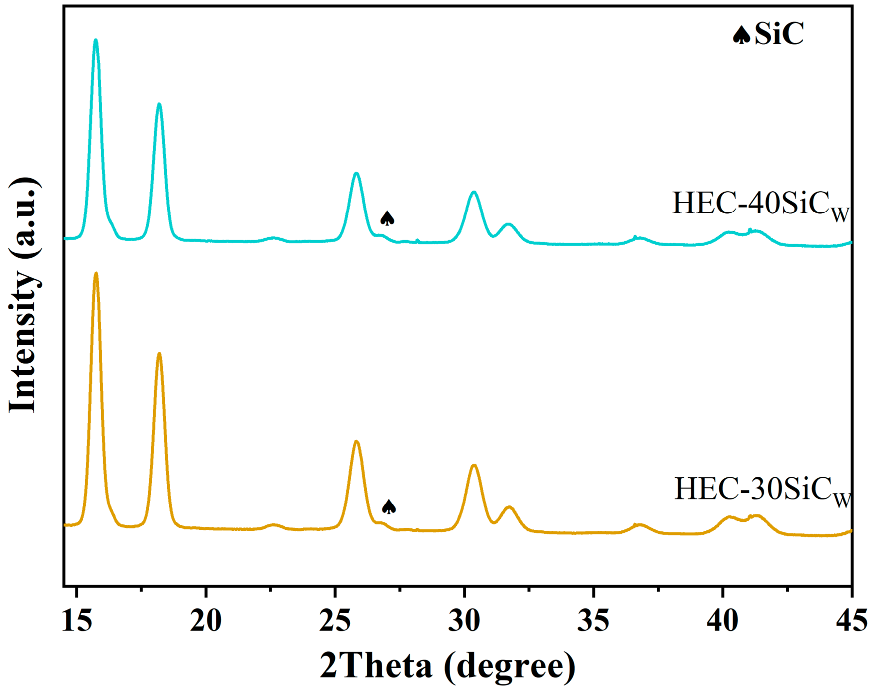

Figure S2. XRD patterns of HEC-30SiCw and HEC-40SiCw obtained under the conditions of 5 GPa and 2000 °C

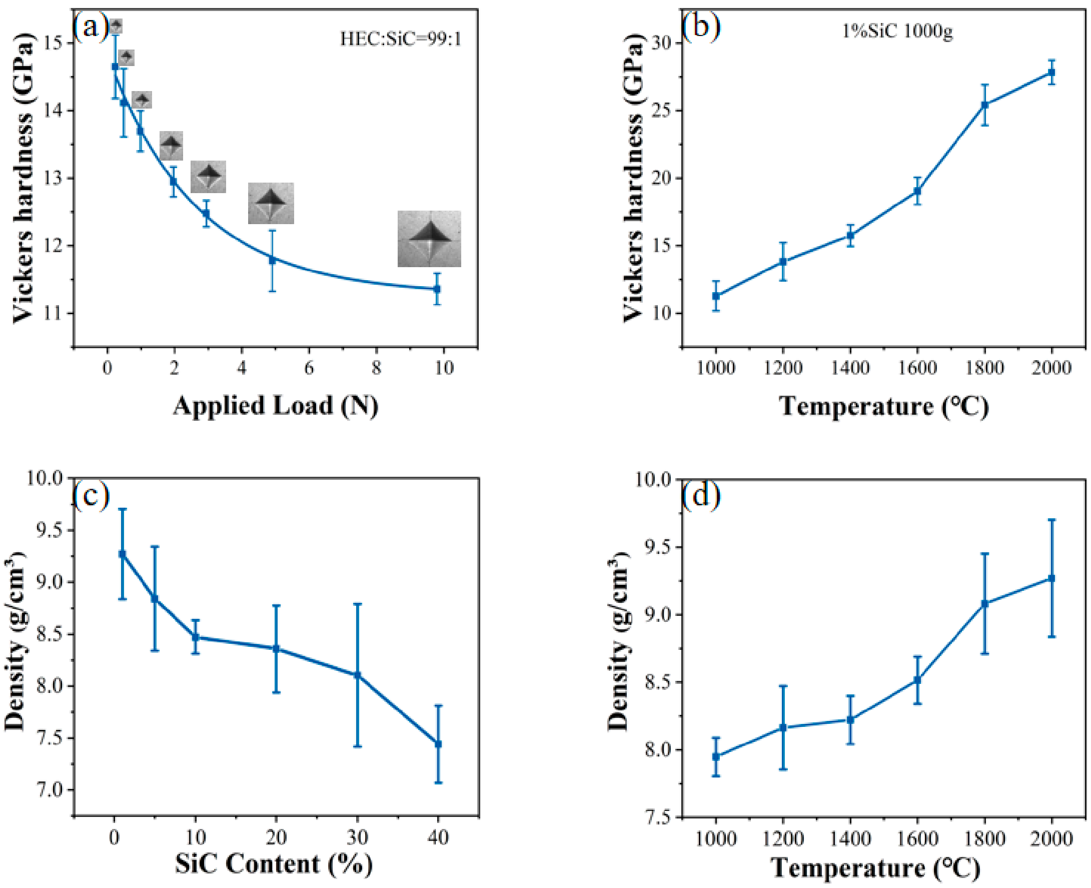

Figure S3. (a) Vickers hardness image of HEC-1SiCw material at 1000°C under different loads. (b) Vickers hardness images at different temperatures at 1kg. (c) Variation of sintering density with silicon addition. (d) Variation of sintered density with sintering temperature for HEC-1SiCw

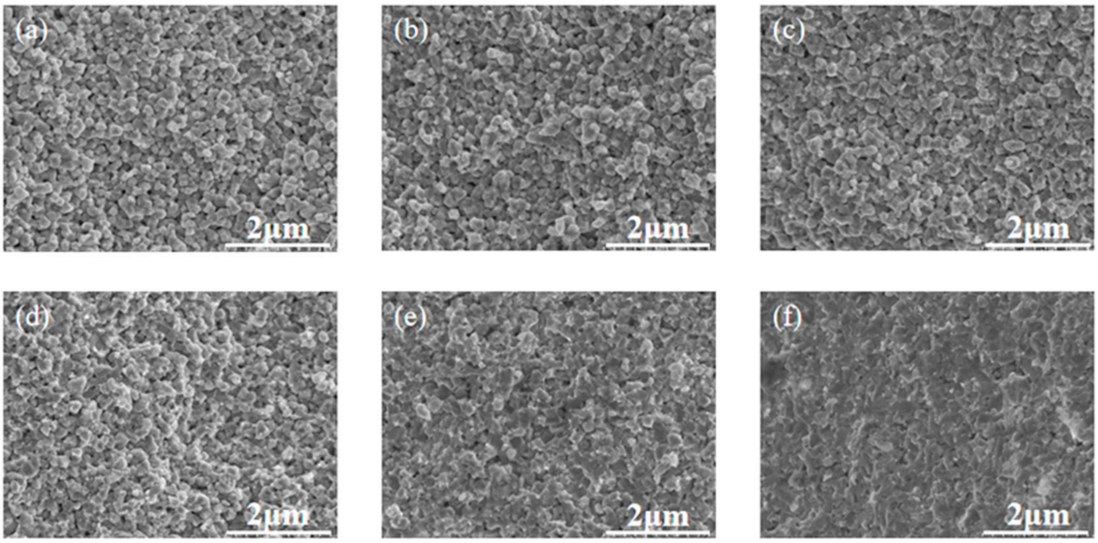

Figure S4. SEM images of fracture surfaces of sintered bodies. (HEC-40SiCw (a) 1000℃, (b) 1200℃, (c) 1400℃, (d) 1600℃, (e) 1800℃, (f) 2000℃).

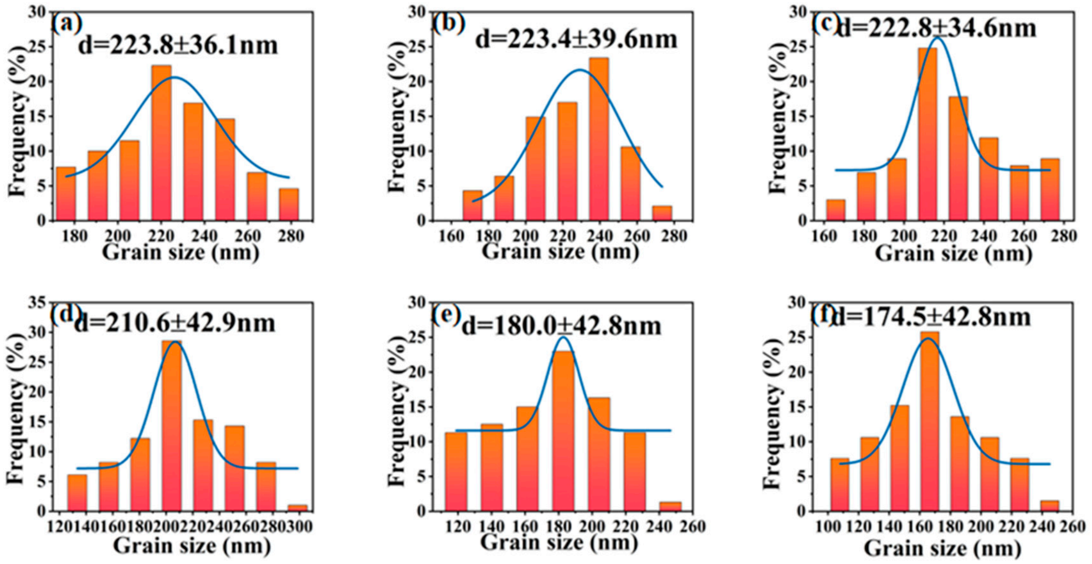

Figure S5. Grain sizes corresponding to different doping ratios of the sintered body. (a) HEC, (b) HEC-5SiCw, (c) HEC-10SiCw, (d) HEC-20SiCw, (e)HEC-30SiCw, (f) HEC-40SiCw.

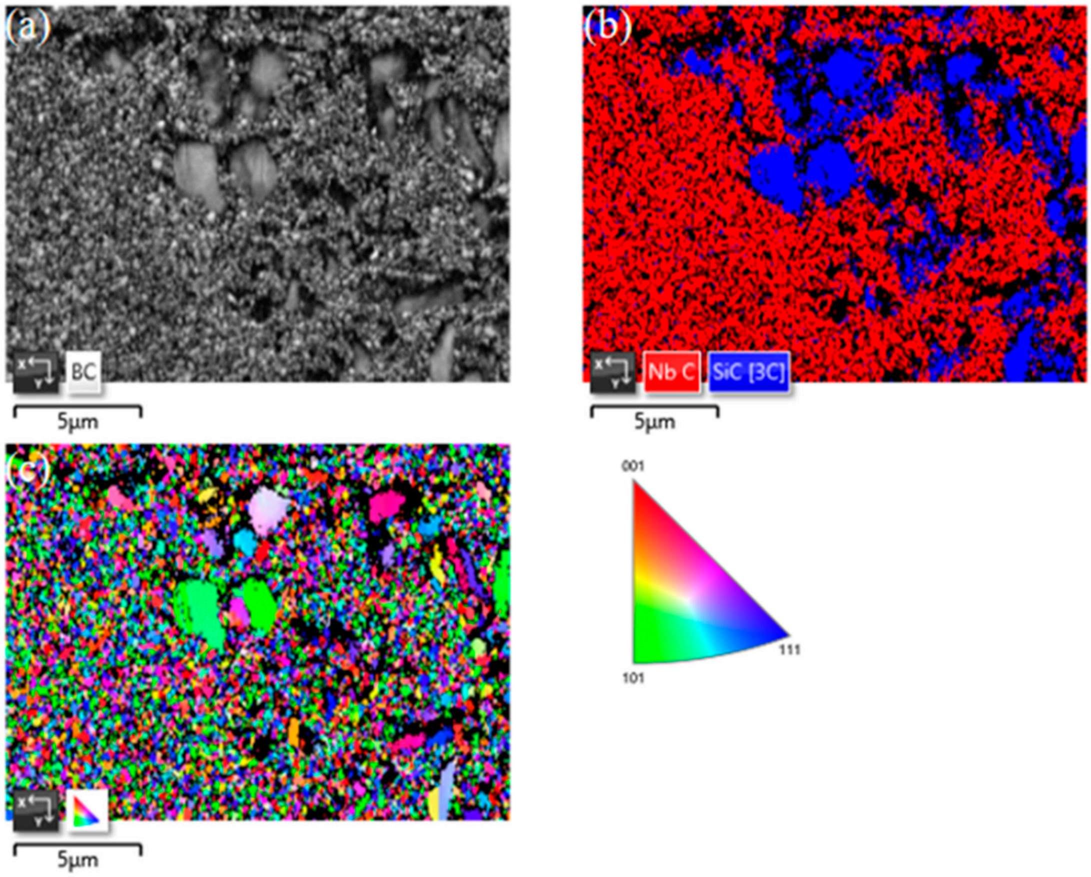

Figure S6. Microstructure of the sintered HEC-SiCw alloy with 40 mol% SiCw under the conditions of 5 GPa and 2000 °C: (a) SEM image, (b) phase diagram, (c) IPF.

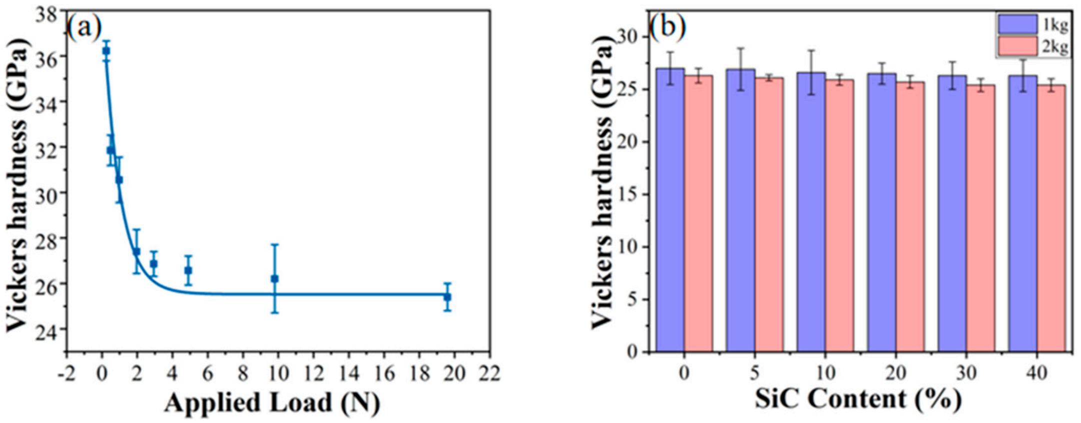

Figure S7. Under the conditions of 5 GPa and 2000 °C (a) Vickers hardness as a function of applied loads, (b) Vickers hardness of different additives at applied loads of 1 kg (10 N) and 2 kg (20 N).

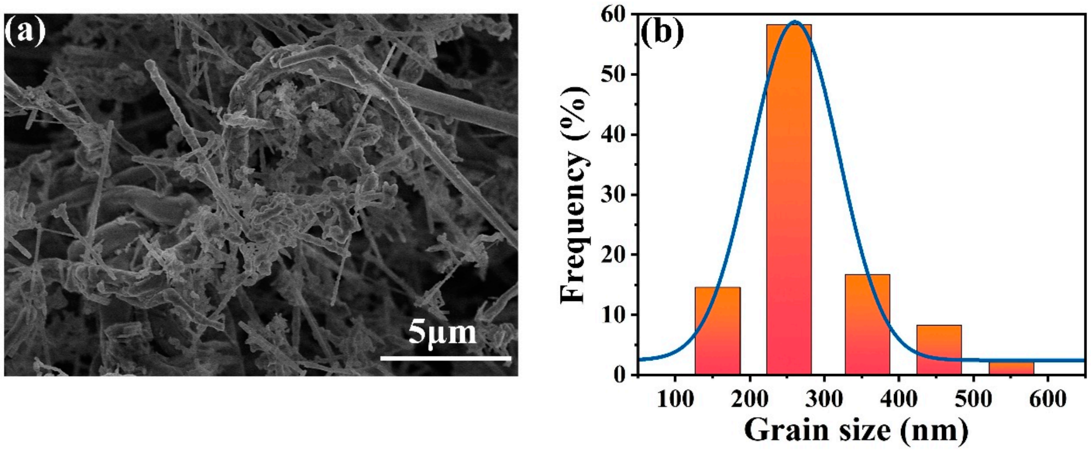

Figure S8. (a) SEM image of the SiC whisker raw material (b) Statistical graph of the grain size
